# 

**Published:** 1883-03

**Authors:** 


### International Medical Congress.

It has been decided to hold the eighth session of the International Medical Congress at Copenhagen, during the days from the 10th to the 16th of August, 1884.

The plan of the organization, and the details of the work of that occasion, are now being arranged, and will be made public in due time.

THE scientific world and the many friends of Professor Owen will be sorry to learn that the state of his health is such as to cause great anxiety.

### EDITORIAL.

### Michigan State Dental Society.

The twenty-eighth annual meeting of this body will be held in Abstract Hall, Detroit, beginning Wednesday, March 28th, at 7:30 P. M., and continue in session two days.

The following programme of subjects for consideration and discussion will be presented viz:

1. Treatment of Deciduous Teeth.
2. Methods of Mounting Artificial Crowns on Natural Roots.
3. Dental Education.
4. Transplanting and Replanting of Teeth.
5. Oral Surgery.
6. Prosthetic Dentistry.

It is strongly urged that there be a full attendance of the members and of the profession of the State generally. And a cordial invitation is extended to the members of other States to be present, and to give their countenance and aid in the good work.

The meetings of this society are always fraught with interest and profit. Let all come.

### Annual Commencement of the Dental Department of the University of Maryland.

The annual commencement exercises of this Dental Department, will be held at the Academy of Music, Baltimore, March 15th, 1883. The members of the dental profession are cordially invited to attend. FERDINAND J. S. GORGAS, M.D., D.D.S., DEAN.

### CONTENTS.

COMMUNICATIONS.

### THE DENTAL REGISTER,

A MONTHLY MAGAZINE DEVOTED TO THE INTERESTS OF THE

**DENTAL PROFESSION.**

*Terms Reduced to $2.00 Per Year. Single Copies, 25 Cents.*

**EDITED BY PROF. J. TAFT, D.D.S.**

AND

***Published by SPENCER & CROCKER, Ohio Dental Depot.***

The volume commences in January and eloses in December.

The REGISTER being issued monthly is always fresh, therefore Indispensable to the practitioner who desires to keep posted up with the new inventions and improvements which are daily developed. Neither expense nor effort will be spared to give value and variety to its contents Articles will be illustrated with well executed engravings whenever they will add to the interest or assist to explanation.

Its extensive circulation and frequency of issue make it one of the BEST DENTAL ADVERTISING MEDIUMS in the United States.

All subscriptions must begin with January or July.

Local or special notices, five lines or less, will be inserted for $3 each insertion; over five lines, 50 cts. per line.

All advertising bills payable in advance, or at furthest, after the issue of the first number containing the advertisement All transient advertisements to be paid for in advance.

☞CONTRIBUTIONS SOLICITED.

Exclusively Editorial Communications should be addressed to the Editor.

The latest number of the REGISTER may be ordered with or without other goods at any time, and all letters should be addressed to

**SPENCER & CROCKER, Ohio Dental Depot, Cincinnati, O.**

